# Probing metabolic states of differentiating stem cells using two-photon FLIM

**DOI:** 10.1038/srep21853

**Published:** 2016-02-25

**Authors:** Aleksandra V. Meleshina, Varvara V. Dudenkova, Marina V. Shirmanova, Vladislav I. Shcheslavskiy, Wolfgang Becker, Alena S. Bystrova, Elena I. Cherkasova, Elena V. Zagaynova

**Affiliations:** 1 Institute of Biomedical technologies, Nizhny Novgorod State Medical Academy, Minin and Pozharsky Square, 10/1, Nizhny Novgorod, 603005, Russia; 2 Institute of Biology and Biomedicine, Nizhny Novgorod State University, Gagarin Avenue, 23, Nizhny Novgorod, 603950, Russia; 3 Department of Radiophysics, Nizhny Novgorod State University, Gagarin Avenue, 23, Nizhny Novgorod, 603950, Russia; 4 Becker & Hickl GmbH Nahmitzer Damm 30, Berlin, 12277, Germany

## Abstract

The ability of stem cells to differentiate into specialized cell types presents a number of opportunities for regenerative medicine, stem cell therapy and developmental biology. Because traditional assessments of stem cells are destructive, time consuming, and logistically intensive, the use of a non-invasive, label-free approach to study of cell differentiation provides a powerful tool for rapid, high-content characterization of cell and tissue cultures. Here, we elucidate the metabolic changes in MSCs during adipogenic differentiation, based on the fluorescence of the metabolic co-factors NADH, NADPH, and FAD using the methods of two-photon fluorescence microscopy combined with FLIM. To estimate the contribution of energy metabolism and lipogenesis in the observed changes of the metabolic profile, a separate analysis of NADH and NADPH is required. In our study we demonstrated, for the first time, an increased contribution of protein-bound NADPH in adipocytes that is associated with lipogenesis. The optical redox ratio FAD/NAD(P)H decreased during adipogenic differentiation, and that this was likely to be explained by the intensive biosynthesis of lipids and the enhanced NADPH production associated with this. Based on the data on the fluorescence lifetime contribution of protein-bound NAD(P)H, we registered a metabolic switch from glycolysis to oxidative phosphorylation in adipocytes.

The ability of stem cells both to self-renew and to differentiate into specialized cell types presents a number of opportunities for regenerative medicine, stem cell therapy and developmental biology. However, effective control of the stem cell differentiation is a great challenge because of the complex relationships between the different signaling pathways, the extracellular microenvironment, and the metabolic requirements of the cell^1^,^2^.

It is known that stem cells possess metabolic characteristics different from differentiated cells. Highly proliferating stem cells have increased needs for energy (ATP) and reducing cofactors as well as for carbon, nitrogen and hydrogen in order to support intensive biosynthesis^3^,^4^. Partial breakdown of glucose through glycolysis and the pentose phosphate pathway provides a compromise between the catabolic generation of ATP and reducing cofactors and the production of biosynthetic substrates to meet the cells’ anabolic requirements^5^. Although glycolysis is less efficient than oxidative phosphorylation, it enables a fast rate of ATP generation.

Differentiated cells no longer need to sustain a high rate of replication and therefore have lower anabolic demands. However, they require large amounts of energy to support cellular homeostasis and their increasingly specialized functions and typically produce ATP through oxidative phosphorylation^6^.

Multiphoton fluorescence microscopy and fluorescence lifetime imaging (FLIM) are powerful techniques for the non-invasive characterization and long-term monitoring of the functional changes that underlie cellular metabolism^7^,^8^. The possibilities for investigating stem cell metabolism in normal and pathological conditions *in vitro* and *in vivo* using these methods have been demonstrated widely, in ref. 9, 10, 11, 12, 13.

Metabolic imaging is generally based on analysis of the reducing cofactors nicotinamide adenine dinucleotide NAD(P)H and flavin adenine dinucleotide FAD^14^,^15^,^16^.

The ratio of their fluorescence intensities (FAD/NADH or FAD/(NADH+FAD), referred to as the optical redox ratio, is commonly used to estimate the metabolic profile of a cell or tissue. If oxidative phosphorylation is enhanced in cells, the oxidation of NADH to NAD^+^ and FADH2 to FAD+ is prevalent over the glycolytic reduction of NAD+ to NADH, and so the redox ratio increases^17^. However, this may only be evident if no other metabolic pathways contribute to the concentrations of the fluorescent cofactors.

The NAD/NADH and NADP/NADPH redox couples are the major determinants of the emphasis of cellular metabolism. NAD drives ATP production in the cytosol by glycolysis, and in the mitochondria by oxidative phosphorylation, while its phosphorylated analogue NADP governs the lipid, amino acid and nucleotide biosynthetic pathways and the defense against reactive oxygen species (ROS) by glutathione^18^. Metabolic pathways related to cell differentiation are known to change NAD(P)H binding sites, and enzymatic binding is directly related to NAD(P)H cycling through the energy production pathway^19^.

It is known that any fluorescence signal coming from NAD(P)H is composite and contains contributions from free and bound NADH and bound NADPH^17^. Previous studies have revealed that NAD(P)H fluorescence consists of short- and long-lifetime components corresponding, respectively, to the free and protein bound forms^20^. Protein-bound NAD(P)H is characterized by a complex multi-exponential lifetime decay that has been related to its binding to different enzymes, such as malate dehydrogenase (MDH) and lactate dehydrogenase (LDH)^21^. In a recent work, Blacker *et al.* (2014) demonstrated that FLIM can differentiate quantitatively between NADH and NADPH; however this finding has not, as yet, been explored in metabolic studies^22^.

Adipogenic differentiation of mesenchymal stem cells (MSCs) is associated with two parallel metabolic processes – the shift of cellular energy metabolism from glycolysis to oxidative phosphorylation and the activation of lipogenesis. We proposed that application of a three-exponential fitting model during FLIM of NAD(P)H could allow us, simultaneously, to distinguish between NADH and NADPH and to investigate the changes in energy metabolism (by protein-bound NADH) and the activation of lipid biosynthesis (by protein-bound NADPH). Although the adipogenic differentiation is accompanied by oxidative stress, and NADPH is involved in the antioxidant replenishment^23^, this process was out of the scope of this work.

The aim of the present work was to study metabolic changes in MSCs during adipogenic differentiation, based on the fluorescence of the metabolic co-factors NADH, NADPH, and FAD. Cellular metabolism was examined by monitoring the optical redox ratio (FAD/NAD(P)H), the fluorescence lifetime contributions of the free and bound forms of NADH and the bound form of NADPH. Two-photon fluorescence microscopy combined with FLIM was used to analyze this fluorescence in living cells.

## Results

### Adipogenic differentiation of MSCs

**Figure 1:**
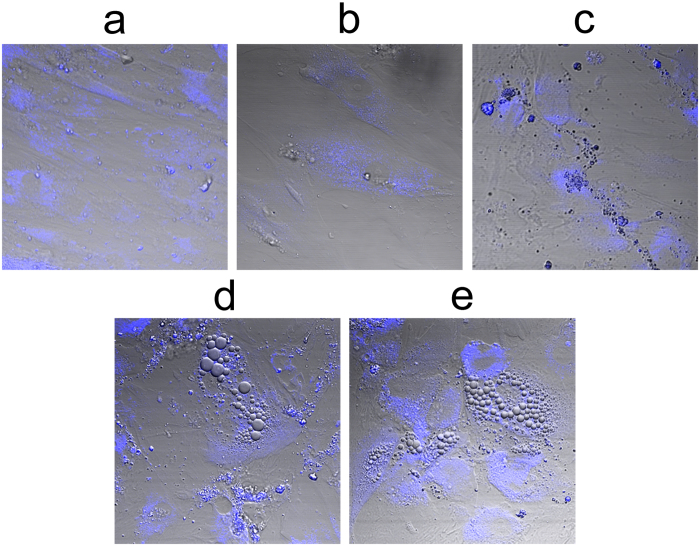
Adipogenic differentiation of MSCs. Fluorescence NAD(P)H is shown inblue, transmitted light – in gray. (**a–e**) - 0, 5, 12, 19, 26 days of differentiation respectively For NAD(P)H: excitation - 750 nm, detection - 455–500 nm. The image size is 213 × 213 μm (1024 × 1024 pixels).

MSC differentiation was assessed by noting morphological changes and the development of lipid vacuoles. On days 5 and 12 of differentiation the MSCs had a spindle-shaped morphology; the cell population was homogeneous. By days 19 and 26 the differentiated cells had become polygonal in morphology and contained a great number of lipid vacuoles (Fig. 1). The characterization of the differentiated cells is presented in the Table 1.

### Assessment of the metabolic status of MSCs during adipogenic differentiation using their optical redox ratio

To estimate the general level of metabolic activity of the cells during adipogenic differentiation, the fluorescence intensities of NAD(P)H and FAD were measured and represented as their redox ratio (FAD/NAD(P)H). The redox ratio was calculated for undifferentiated MSCs (days 0, 5, 12) and for differentiated cells at other stages of differentiation (days 19 and 26).

**Figure 2:**
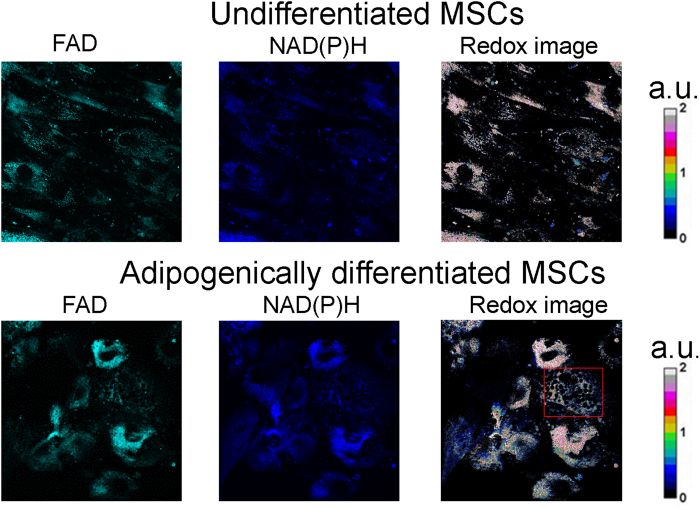
Fluorescence and optical redox images (fluorescence intensity of FAD/NAD(P)H) of undifferentiated MSCs (day 0) and adipogenically differentiated MSCs (day 26). Fluorescence of FAD is shown in green, fluorescence of NAD(P)H - in blue. For NAD(P)H: excitation - 750 nm, detection - 455–500 nm, for FAD: excitation - 900 nm, detection - 500–550 nm. The image size is 213 × 213 μm (1024 × 1024). Adipogenically differentiated MSCs are shown by red square.

**Figure 3:**
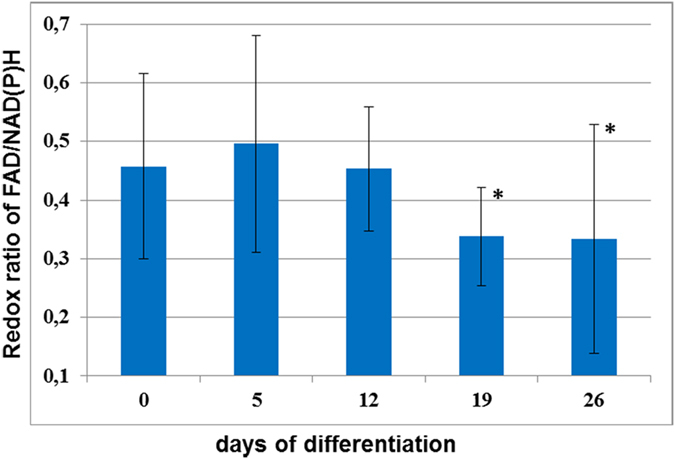
The dynamic of the redox ratio (fluorescence intensity of FAD/NAD(P)H) in undifferentiated MSCs (day 0 and 5) and cells at all stages of differentiation, mean ± SD. *statistically significant difference with “0 day differentiation”, p < 0.05.

No significant changes in the redox ratio values were detected until 19 days after the induction of differentiation. On days 19 and 26 a decrease in the redox ratio could be observed (Fig. 2). The dynamics of the redox ratio during adipogenic differentiation is illustrated in Fig. 3. The changes in the co-factor fluorescence and, consequently, in the redox ratio for differentiated adipocytes may be associated with both energy metabolism and the biosynthesis of lipids. Since a metabolic shift to oxidative phosphorylation is expected during the differentiation of stem cells and that would lead to an increase in the redox ratio, we speculated that the observed decrease of the ratio resulted from the accumulation of NADPH in the process of lipogenesis (oxidative stress cannot be excluded though).

### Proportions of NADH and NADPH during adipogenic differentiation

To study cellular energy metabolism and fatty acid synthesis during adipogenic differentiation, we analyzed the fluorescence lifetime contributions of the free and protein-bound forms of NADH and NADPH.

Using liquid chromatography/tandem mass spectrometry (LC/MS-MS), Quinn K.P. *et al.* (2013) had previously demonstrated that the intracellular concentration of NADH was 33.7 ± 13.1 fold higher than the concentration of NADPH^24^.

In 2014, Blacker *et al.* were the first to use FLIM for the separation of NADH and NADPH fluorescence in live cells (HEK293) and tissues (mammalian cochlea).

Using numerical methods in combination with biochemical manipulations on the cells, the intracellular fluorescence lifetimes of NADH and NADPH were evaluated to be 1.5 ± 0.2 and 4.4 ± 0.2 ns, respectively^22^.

Since the differentiation of the MSCs may result in changes in the fluorescence lifetimes and the amplitudes of the free and the two bound forms of NADH, it would be natural to use the three fluorescence lifetime components in a fitting model of the fluorescence decay.

While for a single exponential fit, one hundred photons per pixel may be sufficient, for a three exponential fit one would need two orders of magnitude more photons per pixel to obtain reasonable accuracy for the fluorescence lifetime. This could be achieved either through long image acquisition times, or by using high laser power incident on the sample. As both approaches may result in photobleaching they cannot be considered as options in the case of experiments with differentiating SCs. Alternatively, binning of the pixels can give the required photon statistics, however this would be at the expense of the spatial resolution. In addition, if the sample is very heterogeneous the binning may result in erroneous calculations of the fluorescence lifetimes. The way around the problem is to use *a priori* knowledge on the lifetimes of the phosphorylated and unphosphorylated components.

The fixing of these lifetimes significantly relaxes the requirements on the minimum photon numbers and speeds up the computational times. It is reasonable to hypothesize that conclusions on the fluorescence lifetimes of NADH and NADPH can be extended to all cell types, as the fluorescence lifetime of NAD(P)H when bound to an enzyme is determined by its local environment in the binding site^25^, and the NADH and NADPH-binding sites are two of the most highly conserved in all biology^26^.

**Figure 4:**
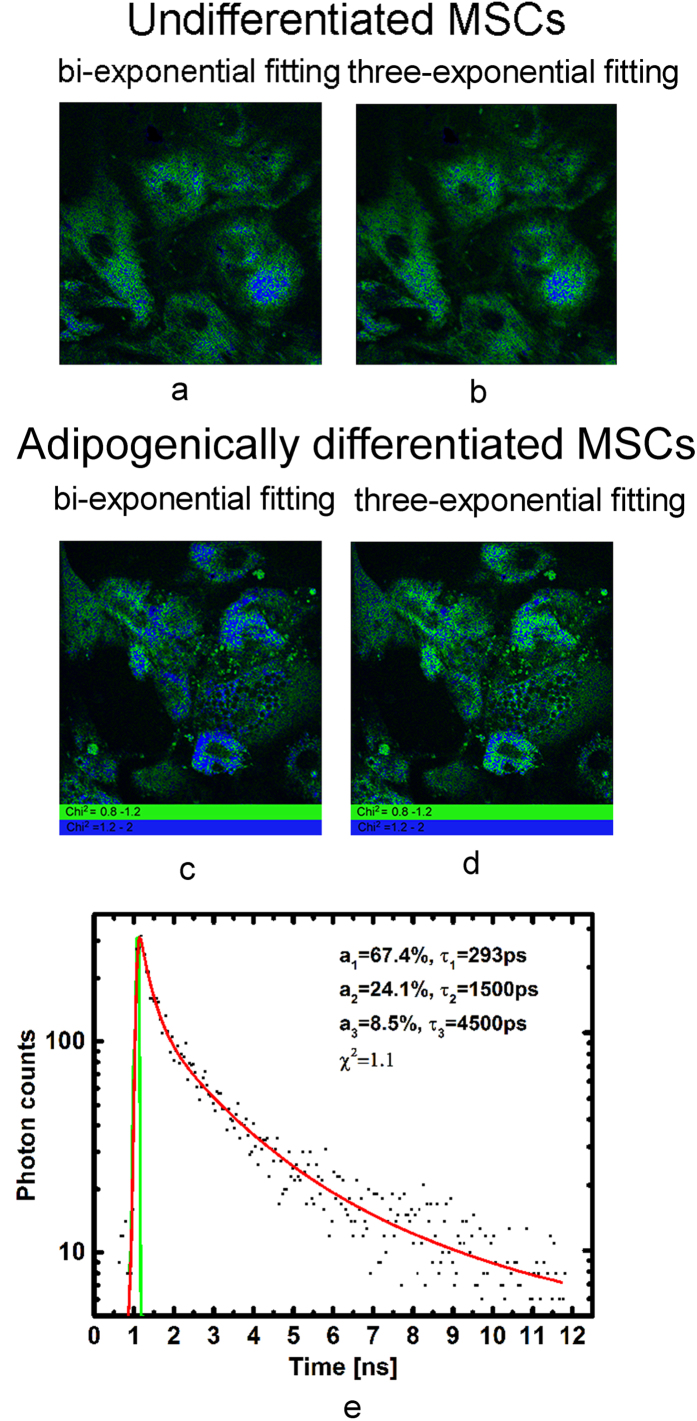
(**a,c**) The distribution of χ^2^ for bi-exponential fitting model for undifferentiated and differentiated MSCs, respectively. (**b,d**) The distribution of χ^2^ for three-exponential fitting model for for undifferentiated and differentiated MSCs, respectively. The value of χ^2^ from 0.8 to 1.2 is shown in green, from 1.2 to 2.0 - in blue. Field of view for all the images is 213*213μm (512*512 pixels) with 256 time channels. (**e**) Example of three-exponential fit in the specific spot on the sample. Black dots: experimental data; red curve: fit; green curve: computed instrument response function. The bi-exponential fit results in χ^2^ = 1.23 in the same spot.

**Figure 5:**
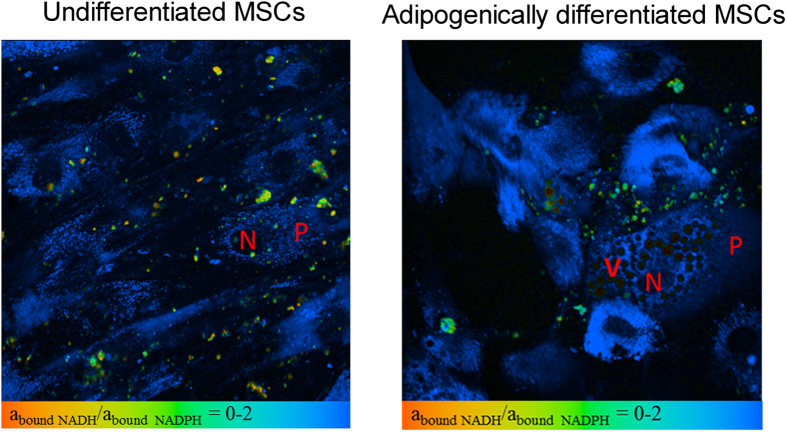
The distribution of NADH and NADPH in undifferentiated (day 0) and adipogenically differentiated MSCs (day 26). Pseudocolor-coded images of the a_boundNADH_/a_boundNADPH_ ratio. V- vacuoles, P- perimeter of cells, N-nucleus. Field of view 213*213μm (512*512 pixels).

Therefore the NADH and NADPH fluorescence lifetimes were fixed at 1.5 and 4.4 ns, respectively, enabling an analysis of the goodness of fit (χ^2^) across the images to be performed (Fig. 4A–E). For visualization of the comparative concentrations of NADH and NADPH we imaged the relationship of the amplitudes corresponding to each coenzyme (a2/a3) (Fig. 5). Thus, we could identify areas with different concentrations of the phosphorylated and nonphosphorylated forms of NADH. The distributionof χ^2^ with good rateson the cells area was also assessed.

We showed that the use of three-exponential fit improves the relative square of χ^2^ (0.8–1.2) by 6.8% in undifferentiated MSCs and by 17.1% in adipocytes when compared with the bi-exponential fit. The greater percentage of good χ^2^ area in differentiated cells potentially testifies to higher NADPH production. The spatial distribution of χ^2^ (0.8–1.2) within the cells for the bi- and three-exponential fittings is demonstrated in Fig. 4A–D. Figure 4E illustrates example profile of NADH fluorescence decay, including measurement data, modelled data (red curve), generated instrument response function, and associated fit values computed with SPCImage software. While double-exponential model yielded satisfactory residual profiles (not shown), the three component model consistently resulted in smaller fitting errors.

Therefore, comparison of the bi- and three-exponentialfitting models for NAD(P)H analysis in MSCs differentiation showed no differences in their χ^2^ values, with a greater area of good rates of χ^2^ (0.8–1.2) in a cell for the three-exponential fit. Taking this into account, we found the three-exponential fitting model to be appropriate for estimating NADH and NADPH, separately, during adipogenic differentiation.

As, during the differentiation of stem cells, the energy metabolism switches from glycolysis to oxidative phosphorylation, we suggested that the fluorescence contribution from the free form of NADH should decrease, while that from the protein-bound form of NADH should increase. At the same time *de novo* fatty acid synthesis and oxidative stress proceed with the participation of NADPH, so we hypothesized that the differentiation of MSCs into adipocytes should be accompanied by an increase in the NADPH contribution to the decay of NAD(P)H fluorescence. Using the three-exponential fitting model we were able to separate the bound forms of NADH and NADPH and estimated the fluorescence lifetime contributions of free NADH and the protein-bound NADH and NADPH.

**Figure 6:**
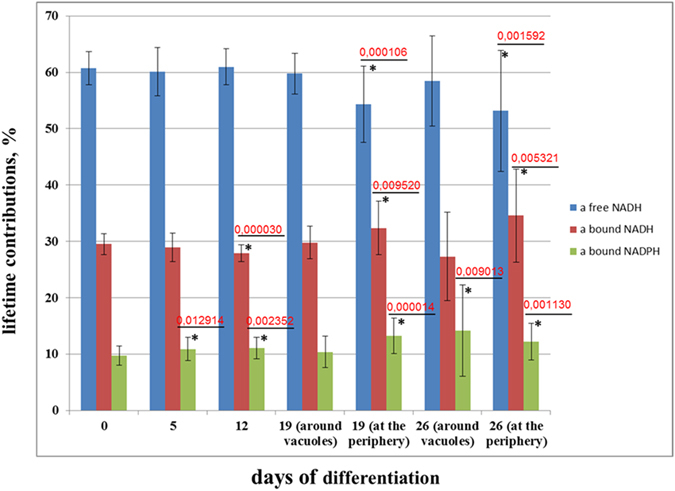
The dynamic of lifetimes contributions of free NADH, and protein-bound forms of NADH and NADPH in undifferentiated MSCs (days 0 and 5) and cells at all stages of differentiation. mean ± SD. *statistically significant difference with day 0. P values are shown.

It was found that the contribution of free NADH (a_freeNADH_) did not change up to day 19. A statistically significant decrease in the short fluorescence lifetime contribution was shown in differentiated cells (days 19 and 26) in the regions of cytoplasm at the cell periphery as compared with undifferentiated MSCs (day 0) (Fig. 6).

The reverse trend was observed in the contribution of bound NADH (a_boundNADH_) on days 19 and 26. It is interesting that the fluorescence lifetime contribution of bound NADH increased only in the regions of cytoplasm at the periphery of the cells. The statistically significant decrease in the contribution of the bound form of NADH on day 12 of differentiation is probably associated with the redistribution of bound NADH to bound NADPH (Fig. 6).

The contribution of bound NADPH (a_boundNADPH_) was higher at all time-points of adipogenic differentiation when compared with undifferentiated MSCs. The maximum elevation of bound NADPH was detected in differentiated cells (days 19 and 26) without, however, any differences between the zones of the cytoplasm at the cell periphery and around the lipid vacuoles (Fig. 6).

Therefore, the FLIM data, processed with the three-exponential fitting model,on the increase in the contribution of protein-bound NADH in the cytoplasm at the cell periphery testify to the metabolic switch from glycolysis to oxidative phosphorylation during the differentiation of stem cells. The rise in the contribution of bound NADPH is probably associated with the biosynthesis of lipids.

We also compared the ratio a_boundNADH_/a_boundNADPH_ in undifferentiated MSCs and adipocytes. It can be seen from Fig. 5 that the distribution of the a_boundNADH_/a_boundNADPH_ ratio in adipocytes is inhomogeneous, varying from 0 in the areas of the cytoplasm next to lipid vacuoles (which indicates a contribution to the ratio only from NADPH), to 2 in the other parts of the cytoplasm.

Therefore, these results showed an increase in the NADPH concentration in differentiated cells owing to the activation of fatty acid biosynthesis during adipogenic differentiation.

## Discussion

In this study we investigated the metabolic changes in living MSCs during adipogenic differentiation, using two-photon fluorescence microscopy and FLIM. Both energy metabolism and the biosynthesis of lipids were addressed. Based on the data on the fluorescence lifetime contribution of protein-bound NAD(P)H, we registered a metabolic switch from glycolysis to oxidative phosphorylation in adipogenically differentiated MSCs. We demonstrated, for the first time, an increased contribution of protein-bound NADPH in adipocytes associated with lipogenesis at least. We also found that the optical redox ratio FAD/NAD(P)H decreased during adipogenic differentiation, and that this was likely to be explained by the intensive biosynthesis of lipids and the enhanced NADPH production associated with them. Besides, NADPH is involved in a number of pathways, including antioxidant replenishment through the GPX pathway. It is known that differentiation processes (adipogenesis and osteogenesis) is regulated by ROS. Mitochondrial complexes I and III, and the NADPH oxidase isoform NOX4 are major sources of ROS production during MSC differentiation. NOX enzymes generate ROS by oxidizing intracellular NADPH to NADP+ and the transfer of electrons through membranes to reduce molecular oxygen and generate the superoxide anion as a primary product. The adipogenic process increases the expression of antioxidants^23^. Although oxidative stress can also contribute to the change in the optical redox ratio and NADPH, observed in our study, we suppose that the main contribution was from lipogenesis, that manifested itself in appearance of a great number of lipid vacuoles in differentiated cells.

It is known that energy metabolism is an important regulator of the ability of stem cellsto undergo both differentiation and self-renewal^26^,^27^,^28^. Currently, there exists a range of methods to study the metabolism of stem cells, such as electron microscopy, immunohistochemistry and colorimetric metabolic assays. However, these methods are invasive, require the introduction of exogenous labels into the cells and do not provide complete information about the physiological changes in the living cells during their growth, proliferation and differentiation. In contrast, multiphoton fluorescence microscopy combined with FLIM is an emerging modality that permits repeated non-destructive measurements of the components within living cells.

In the fluorescence intensity measurement scheme, NAD(P)H is often paired with another coenzyme in oxidative metabolism, FAD^10^. As FAD and NADH are the only forms of electron carriers that exhibit fluorescence, their fluorescence intensity ratio is generally used to estimate the metabolic profile of a cell or tissue.

A number of studies have demonstrated dynamic changes in the redox ratio in stem cells undergoing osteogenic, adipogenic and chondrogenic differentiation^24^,^29^,^30^. However, the changes in the redox ratio during differentiation display opposite trends. There are studies that show a decreased redox ratio during adipogenic differentiation^8^,^24^,^29^. However, an increased ratio during osteogenic differentiation was detected in ref. 8.

Our results are consistent with the data indicating a decreased redox ratio in metabolically active, differentiated cells. We associate the decreased ratio with an increased NAD(P)H concentration, rather than with a decreasing concentration of FAD. Croce *et al.* (2007) also demonstrated an increased NADH concentration in cells with higher metabolic rates, while others have demonstrated a decrease in NADH fluorescence associated with increased metabolic activity^31^,^32^.

The optical redox ratio is sensitive to the balance between the rates of ATP consumption and glucose catabolism in a cell. Undifferentiated MSCs produce ATP primarily through glycolysis, and this is accompanied by NADH production, while those undergoing differentiation display a shift towards oxidative phosphorylation for their energy needs. It is generally accepted that glycolytic cells show decreased redox ratios, while in oxidative cells the ratio increases^6^.

In the case of stem cells undergoing adipogenic differentiation the situation is complicated by the intensive biosynthesis of lipids, where NADPH is involved. Similar to our work, Quinn *et al.* showed a decrease in the optical redox ratio associated with lipogenesis during adipogenic differentiation^24^. They suggested that adipogenic differentiation, at least *in vitro*, is associated with an increase in flux through the metabolic pathways using enzymes expressed in the mitochondria. Citrate can then be shuttled out of the mitochondria and cleaved in the cytosol so that acetyl-CoA can be used as a carbon supply for fatty acid synthesis. This process would require the conversion of NAD^+^ to NADH by the LipDH-containing pyruvate dehydrogenase complex (PDHC). Carbon storage in lipid droplets through *de novo* fatty acid synthesis would probably not occur when substantial ATP production is necessary, and consequently the reduced form of NADH may accumulate. The remaining cytosolic oxaloacetate can be converted to pyruvate by malic enzyme (during which conversion, NADPH is also produced) for use in long chain fatty acid synthesis^29^.

However, the optical redox ratio gives only general information about changes in the relative concentrations of the metabolic cofactors, while the specific mechanisms underlying the changes in the metabolic state of the cells remain unclear.

To estimate the contribution of energy metabolism and lipogenesis in the observed changes of the metabolic profile, a separate analysis of NADH and NADPH is required. To separate NADH and NADPH in differentiating stem cells we used FLIM with a three-exponential fitting of the fluorescence decay curves, using the method developed by Blacker *et al.*^22^.

Our results about lower contribution of NADPH compare to NADH to overall fluorescence of NAD(P)H are consistent with other studies. Blacker *et al.* showed that the concentration of bound NADPH decreased ~3-fold in HEK293 cell lines by combining of the data on the enzyme-bound NAD(P)H fluorescence lifetimes measured in the NADK+ and NADK- cells, the quantified [NADH] and [NADPH] values in cell line and a mathematical model in which NADH and NADPH were assumed to possess discrete and distinct fluorescence lifetimes when bound inside the cell. Quinn *et al.* demonstrated on pancreatic islet and porcine heart that the intracellular concentration of NADH was 33.7 ± 13.1 fold higher than NADPH^24^. Klaidman *et al.* showed that the concentration of the fluorescent reduced NADH was 5 times greater than that of the fluorescent NADPH in mouse hippocampus^33^. Moreover, the enhancement of mitochondrial NADH quantum yield due to environmental effects has been estimated to be a factor of 1.25–2.5 greater than that of NADPH^34^. Protein binding can also affect the fluorescence quantum yield of NADH, with up to a 10-fold increase in the fluorescence intensity of mitochondrial protein bound NADH relative to free NADH^35^.

We detected, simultaneously, an increased contribution of protein-bound NADH and a decreased contribution of free NADH, in regions of the cytoplasm at the peripheries of cells in the process of adipogenic differentiation, that probably testifies to a switch of their metabolic pathways to oxidative phosphorylation. This result is in agreement with other studies. For example, Konig K. showed an increase in the bound forms of NAD(P)H during the differentiation of stem cells intoadipocytes^36^, Stringari C. *et al.* demonstrated a higher free/bound NADH ratio in undifferentiated neural progenitor and stem cells than in differentiated neurons, and that stem cells followed a metabolic trajectory from a glycolytic phenotype to an oxidative phosphorylation phenotype through the different stages of differentiation^37^.

Beyond this, we have now shown a rise in the contribution of bound NADPH in undifferentiated cells shortly after the induction of differentiation, before any pronounced changes have appeared in the morphological phenotype, as is associated with the active biosynthesis of lipids at all stages of differentiation, but which is also seen in adipocytes. To the best of our knowledge, the changes of the contribution of bound NADPH in MSCs during adipogenic differentiation have never previously been measured using FLIM with three-exponential fitting of the fluorescence decay curves. However use contribution of bound NADPH and NADH for the study of phenotypic changes requires further investigation and appropriate metabolic measurements.

## Materials and Methods

### Stem cell culture and adipogenic differentiation

All procedures were conducted according to N. K. Koltzov Institute of Developmental Biology Ethic Committee approval.Patients with no notable pathologic history were chosen for this study. Human bone marrow mesenchymal stem cells (MSCs) were isolated from bone marrow of normal donors with informed consent according to the Institutional guidelines under the approved protocol. The MSCs were isolated as described previously^38^. The cells had a fibroblast-like morphology and a typicalMSC phenotype.

The MSCs were cultured in MesenCult™ MSC Basal Medium (Human) (STEMCELL TECHNOLOGIES, Canada) supplemented with 10% fetal bovine serum (FBS) (Hyclone), 0.58 mg/ml L-glutamine (PanEco) and40 U/ml gentamicin. The cell culture was maintained at 37 °C in a 5% CO_2_, humidified atmosphere.

Differentiation was induced by incubating the MSCs in MesenCult™ Adipogenic Differentiation Medium (Human) (STEMCELL TECHNOLOGIES, Canada).The medium was replaced every 3–4 days over the experimental a period of up to 4 weeks.

For microscopic imaging, 4*10^5^ cells were transferred into a sterile dish with a cover-glass bottom (0.17-mm thick) and incubated for one day until they attached to the glass surface. Cell monolayers were used.

The cells were imaged before the induction of differentiation (day 0) and on days 5, 12, 19 and 26 subsequently. The cells were washed twice using phosphate-buffered saline, and then placed in FluoroBrite™ DMEM (Gibco) with 10% FBS and 0.58 mg/ml L-glutamine (PanEco) and 40 U/ml gentamicin.

Morphological changes were assessed by counting the number of cells with spindle-shaped and polygonal morphology at each differentiation stage. The average numbers of differentiated cells and vacuoles, and the area of vacuoles (in pixels and %) in each differentiated cell were calculated on 19 and 26 days. The cells were counted in 10 to 20 fields of view. All the measurements were taken using ImageJ 1.39p software (NIH, USA).

### Multiphoton fluorescence microscopyand FLIM

The two-photon exited fluorescence intensity images of NAD(P)H and FAD, together with the FLIM images of NAD(P)H were obtained on an LSM 710 (Carl Zeiss, Germany) inverted laser scanning confocal microscope equipped with a time-correlated single photon counting (TCSPC) system (Simple-Tau 152, Becker&Hickl GmbH, Germany).

NAD(P)H and FAD fluorescence was excited with a Chameleon Vision II (Coherent, USA) Ti:Sa femtosecond laser, using an 80 MHz repetition rate and a pulse duration of 140 fs at wavelengths of 750 nm and 900 nm, respectively. Emission was detected in the ranges 455–500 nm for NAD(P)H, and 500–550 nm for FAD.

An average of 8000–10000 photons were assessed per decay curve. The absence of photobleaching was confirmed by the constant photon count rate during image acquisition.

The average power of the Ti:Sa laser was measured using a PM100A power meter (ThorLabs Inc., USA). An internal microscope reference, reporting the two-photon excitation efficiency, was used to controlthe laser power in all the experiments^39^,^40^. The average power incident on the samples was ~6 mW.

A С-Apochromat 40x/1.2 water immersion objective wasused for image acquisition. For the assessment of morphological changes in the MSCs during differentiation the images were obtainedin the transmitted channel.

During the experiments the cells were maintained at 37 °C and 5% CO_2_in an XL multi S Dark LS incubator (PeСon GmbH, Germany).

Two photon excitation, when used at a reasonably low excitation power, is safe to live cells and tissues, as has been shown in ref. 41, 42, 43, 44, 45, 46. Invasive effects are smaller than for one-photon excitation because there is virtually no excitation in out-of-focus planes. The TCSPC FLIM technique used in our experiments provides near-ideal photon efficiency, and uses more efficient detectors^47^. The excitation dose is therefore lower than in the early experiments, and so are possibly adverse effects on the cells. TCSPC FLIM has been already been used in stem cell imaging^48^, and even in clinical applications^44^,^49^, and in a large number of other live cell and tissue experiments^47^.

### Optical redox ratio calculation

The optical redox ratio is defined as the fluorescence intensity of FAD divided by the fluorescence intensity of NAD(P)H^50^. The redox ratio was calculated from corresponding two-photon fluorescence images of FAD and NAD(P)H after subtracting the backgroundon a pixel by pixel basis using ImageJ 1.39p software (NIH, USA).

### Fluorescence lifetime analysis

SPCImage (Becker&Hickl) software was used to fit the fluorescence signal at each area of 3 × 3 pixels (bin 1), or 25 × 25 pixels (bin 2), depending on the photon statistics, to both double- and triple-exponential decay according to the following equation:


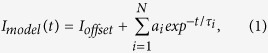


where *N* = 2 or 3 and 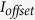 represents the noise coming from a detector and ambient light; 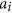 and 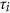 represent the amplitude (or relative contribution), and fluorescence lifetime of component 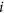, respectively.

The offset has to be taken into account to avoid the artificial generation of a long-lifetime component by the fitting process. The “offset” signal 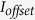
can either be measured by an independent dark count measurements experiment or determined automatically by means of the photons which are in the time channels at the end of the falling part of the fluorescence decay trace. We used the latter approach.

Equation (1) is valid if the width of the instrument response function (IRF) of the measurement system is extremely small compared to the width of the time channels of the histogram in which the photons are stored. The IRF defines the overall time-resolution of the measurement system. It is built up by the pulse-width of the laser (which is negligible small for a femtosecond laser system), the electrical resolution of the TCSPC card and (most important) the transit-time-spread of the detector. In a typical experiment (fast lifetimes, small channel-width) the measured intensity follows a mathematical convolution of the model function 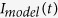 and the instrument response function 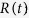



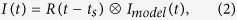


Here the calculated function which is fitted to the decay trace is an integral over time. The SPCImage software provides an “estimation” of this response function by calculating the first derivative of the rising part of the fluorescence. The parameter 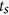
denotes a linear shift between the response function and the fluorescence and is determined automatically by the software.

The average lifetime for each binned area with double or triple-exponential fits was calculated using the formula:


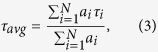


The fluorescence lifetimes and their contributions (free and protein-bound forms of NAD(P)H: a _freeNADH_, a _boundNADH_, a _boundNADPH_) for the areas of interest were calculated by finding the global minimum of the χ^2^ value for three-exponential fittings.

The mean values of χ^2^ in undifferentiated MSCs and in adipocytes were calculated in the same areas of the cells’ cytoplasm for both the bi-exponential and three-exponential fittings. Then the distribution of χ^2^ in the cells was assessed and the area with good rates of χ^2^ (0.8–1.2) relative to the area of the cell cytoplasm was calculated for both fittings. The value of χ^2^ from 0.8 to 1.2 was imaged in green color, from 1.2 to 2.0 - in blue on corresponding images. The fittings of the decay curves have not yielded χ^2^ values from 0 to 0.8 (originally marked by red color).

On days 0, 5 and 12, the fluorescence in the cytoplasm of undifferentiated cells was analyzed, while, only the differentiated cells on days 19 and 26 were considered (regions of the cytoplasm at the cell periphery and around vacuoles were processed separately). We hypothesized, that the lipogenesis should be more active around vacuoles than at the cell periphery. For each time-point 12–23 randomly selected cells were inspected.

### Statistical analysis

For statistical evaluation, 9 samples were used. The data about the number of analyzed cells and ROIs at all stages of differentiation are presented in the Table 2. On the days 19 and 26 only differentiated cells were analyzed. At all other time-points the cells were undifferentiated (Table 2). Statistical analyses were performed using STATISTICA 64 software, version 10 (USA). Mean and standard deviation (SD) values were used to express the data. Differences in the mean values were tested for significance using a student’s t-test (*p* ≤ 0.05).

## Additional Information

**How to cite this article**: Meleshina, A. V. *et al.* Probing metabolic states of differentiating stem cells using two-photon FLIM. *Sci. Rep.* **6**, 21853; doi: 10.1038/srep21853 (2016).

## Figures and Tables

**Table 1:** The characterization of MSCs on 19 and 26 days of differentiation.

	Day 19	Day 26
Number of differentiated cells in the population, %	14	36
Number of lipid vacuoles in a differentiated cell	from 12 to 63	from 16 to 60
The area of vacuoles (diameter more than 1microns), pixels	325 (13 microns^2^)	525 (21 microns^2^)
The area of vacuoles of the total cell area without nucleus, %	12	15

**Table 2:** Number of analyzed MSCs and ROIs.

Days of differentiation	0 day	5 day	12 day	19 day	26 day
Number of analyzed cells	20	23	23	12	22
Number of ROIs	21	82	82	68 around vacuoles, 28 at the periphery	126 around vacuoles, 42 at the periphery
